# Plain Truth to Medical Students

**Published:** 1870-11

**Authors:** 


### Plain Truth to Medical Students.

The medical student of 1870 enters upon a career beset with difficulties, but studded with rewards. The heir of centuries of research and devotion, he has to bear the weight of responsibilities ever increasing, and to uphold a standard which must be borne yet higher, and over steep and difficult paths. A higher national standard of education requires that he shall bring to his special duties the culture benefitting a liberal profession; and the increasing application of exact modes of diagnosis and research in treatment demands, at his hands, an acquaintance with branches of science collateral to medicine, formerly not generally required. On this subject it is right to speak very plainly. Notwithstanding improvements in the standard of preliminary educational requirements, our licensing bodies are perforce at this moment with a second-rate standard of mere schoolboy requirements, which is by no means equal to the just demands of medical science, and which affords a very insufficient guarantee for the future of our profession in this country. The lamentable mediocrity of preliminary acquirements which they accept is the real hindrance to the progress of medicine as a science in this country. It is unnecessary to dissimulate the truth. The miserable inferiority in scientific research, the dearth of original work, the want of exactness, the poverty of physiological investigation, the ignorant impatience of "unpractical detail" which we all have to deplore so much in the mass of professional work at this day, are due to the inadequate preliminary cultivation of our students, to their defective training in scientific method, the small base on which the pyramid of medical lore is made to stand. The solemn deprecation of excessive devotion to microscopic research; the empty sneer at chemical physic; the idle and mischievous disregard of instruments of precision—the sphygmograph, the thermometer, the laryngoscope, the ophthalmoscope—are all the expressions of a Philistine ignorance. There is one enemy against which the English student needs to be earnestly cautioned—it is what he calls his "common sense." It is almost as dangerous at the outset as that nondescript cloak of contented ignorance which often makes him in after life an enemy to science and a danger to mankind, and which he then calls his "experience." As a student, we beseech him to trust to nothing but hard-going laborious cultivated research and study. He cannot be too earnestly warned to fit himself for his work by a thorough mathematical training, by a sound knowledge of languages, by real mental discipline, acquired in a working devotion to natural science. It is on this basis that he must build his clinical aptitude and judgment. The inferiority of English to German medicine is due to the inferiority of preliminary training. We earnestly counsel all students into whose hands these pages will fall to ask themselves how far they are fitted to undertake the career which they they are about to enter.—*British Medical Journal. Boston Medical Journal.*

